# Adaptive signals of flowering time pathways in wild barley from Israel over 28 generations

**DOI:** 10.1038/s41437-019-0264-5

**Published:** 2019-09-16

**Authors:** Chaoju Qian, Xia Yan, Yong Shi, Hengxia Yin, Yuxiao Chang, Jun Chen, Pär K. Ingvarsson, Eviatar Nevo, Xiao-Fei Ma

**Affiliations:** 1 Department of Ecology and Agriculture Research, Key Laboratory of Stress Physiology and Ecology in Cold and Arid Regions, Gansu Province, Northwest Institute of Eco-Environment and Resources, Chinese Academy of Sciences, Lanzhou, 730000 Gansu China; 2 School of Life Sciences, Nantong University, Nantong, 226019 Jiangsu China; 3 Germplasm Bank of Wild Species in Southwest China, Kunming Institute of Botany, Chinese Academy of Sciences, Kunming, 650201 Yunnan China; 4 State Key Laboratory of Plateau Ecology and Agriculture, Qinghai University, Xining, 810016 Qinghai China; 5 Agricultural Genomics Institute at Shenzhen, Chinese Academy of Agricultural Sciences, Shenzhen, 450002 China; 6 College of Life Sciences, Zhejiang University, Hangzhou, 310058 Zhejiang China; 7 Department of Plant Biology, Swedish University of Agricultural Sciences, Uppsala BioCenter, SE-750 07 Uppsala, Sweden; 8 Institute of Evolution, University of Haifa, Haifa, 3498838 Israel

**Keywords:** Population genetics, Genetics

## Abstract

Flowering time is one of the most critical traits for plants’ life cycles, which is influenced by various environment changes, such as global warming. Previous studies have suggested that to guarantee reproductive success, plants have shifted flowering times to adapt to global warming. Although many studies focused on the molecular mechanisms of early flowering, little was supported by the repeated sampling at different time points through the changing climate. To fully dissect the temporal and spatial evolutionary genetics of flowering time, we investigated nucleotide variation in ten flowering time candidate genes and nine reference genes for the same ten wild-barley populations sampled 28 years apart (1980–2008). The overall genetic differentiation was significantly greater in the descendant populations (2008) compared with the ancestral populations (1980); however, local adaptation tests failed to detect any single-nucleotide polymorphism (SNP)/indel under spatial-diversifying selection at either time point. By contrast, the WFABC (Wright–Fisher ABC-based approach) that detected 54 SNPs/indels was under strong selection during the past 28 generations. Moreover, all these 54 alleles were segregated in the ancestral populations, but fixed in the descendent populations. Among the top ten SNPs/indels, seven were located in genes of *FT1* (*FLOWERING TIME LOCUS T 1*), *CO1* (*CONSTANS-LIKE PROTEIN 1*), and *VRN-H2* (*VERNALIZATION-H2*), which have been documented to be associated with flowering time regulation in barley cultivars. This study might suggest that all ten populations have undergone parallel evolution over the past few decades in response to global warming, and even an overwhelming local adaptation and ecological differentiation.

## Introduction

Overwhelming evidence supports the hypothesis that the climate has been changing rapidly since the early twentieth century (IPCC 2001; Jones and Osborn et al. 2001). Under different scenarios of greenhouse gas emission, the mean global temperature is predicted to increase by 1.8–4.0 °C by the end of this century (IPCC 2007a; IPCC 2007b). Such rapid global climate change has become one of the major environmental stressors threatening biodiversity and crop production (Lobell and Schlenker et al. 2011; Osborne and Rose et al. 2013; Thomas and Cameron et al. 2004; Thuiller and Lavorel et al. 2005). Based on 131 studies, ~7.9% of species are predicted to become extinct due to the global climate change by the end of 2050 (Urban 2015), with the situation being even worse in tropic and subtropical areas, where crop yields could decline by 10–20% (Ray and Gerber et al. 2015; Thornton and Cramer 2012). In response to this rapid climate change, plants could adjust their phenotypic variation, such as the allocation of biomass and the phenological phase (Bertin 2008; Jump and Penuelas 2005; Root et al. 2003; Wang and Ottle et al. 2014). In recent decades, a growing body of data has documented a warming spring in the Northern Hemisphere, and many spring events including flowering time in many wild plants now occur earlier than before (Bonsal and Zhang et al. 2001; Robeson 2004; Schwartz and Ahas et al. 2006).

For annual plants, flowering is the most important spring event, and it is critical for plants to have precise control over the time of transition from vegetative growth to reproductive growth to successfully reproduce. Flowering time is known to be affected by various environmental factors, such as temperature, photoperiod, and precipitation (Johansson and Bolmgren et al. 2013; Mouradov and Cremer et al. 2002), and numerous studies have predicted that under global warming, many wild species will flower earlier in the year compared with preceding decades, especially in annual and insect-pollinated plants, suggesting that early flowering is an effective strategy to mitigate the negative effects of climate change, especially to global warming (Bock and Sparks et al. 2014; Brunet and Larson-Rabin 2012; Craufurd and Wheeler 2009; Fitter and Fitter 2002; Franks and Sim et al. 2007; Hovenden and Williams et al. 2008; Miller-Rushing and Primack 2008; Totland 1997). Furthermore, studies in some wild-plant species, such as *Brassica rapa*, *Centaurea cyanus*, and *Boechera stricta*, also indicated that such early flowering is an effective adaptative phenological shift in response to global warming (Anderson and Panetta et al. 2012; Franks and Sim et al. 2007; Thomann and Imbert et al. 2015). To date, with the development of sequencing technologies, as well as novel statistical tools, detailed knowledge has been accumulated about the molecular regulation of flowering times in many model plants (Faure and Higgins et al. 2007; Greenup and Sasani et al. 2010; Griffiths and Dunford et al. 2003; Guo and Li et al. 2015; Johansson and Bolmgren et al. 2013; Lee and Ryu et al. 2013; Mouradov and Cremer et al. 2002; Shindo and Aranzana et al. 2005; Trevaskis and Hemming et al. 2007). Previous studies have documented that >180 genes participated in the flowering time pathways to orchestrate flowering time (Blümel and Dally et al. 2015; Flowers and Hanzawa et al. 2009; Sun and Chen et al. 2014), and the core flowering time pathways are believed to be conserved among short- and long-day plants, as well as among monocot and dicot species (Izawa and Takahashi et al. 2003; Peng and Hu et al. 2015). The two most important pathways regulating flowering time are the photoperiodic and vernalization response pathways, among which the former controls flowering by regulating the input and output of the circadian clock (Flowers and Hanzawa et al. 2009; Mouradov and Cremer et al. 2002; Sun and Chen et al. 2014), whereas the latter mostly relies on sensing temperature (Johanson and West et al. 2000; Michaels and Amasino 2001; Shindo and Aranzana et al. 2005). Signals of environmental change are sensed and integrated by these two pathways, and then are transmitted to the downstream genes *CONSTANS* (*CO*) and *FLOWERING TIME LOCUS T* (*FT*), which induce flowering (Bernier and Périlleux 2005; Corbesier and Vincent et al. 2007). Other studies have also highlighted that the genes *FRI* (*FRIGIDA*) and *FLC* (*FLOWERING LOCUS C*) play important roles in regulating flowering time in plants in response to divergent environments (Werner and Borevitz et al. 2005). Moreover, studies of standing genetic variation in non-model trees, such as aspen, spruce, and oak (Chen and Källman et al. 2016; Chen and Källman et al. 2012; Keller and Levsen et al. 2012; Ma and Hall et al. 2010), have shown that genes from the photoperiodic pathway were involved in local adaptation. Generally speaking, most studies have focused on the genetic mechanisms of flowering time changes between different populations that have been affected by spatial heterogeneity. However, few studies have sampled the same population sets across time periods with dramatically different climatic conditions, which could provide insights into the genetic mechanisms regulating long-term adaptation to shifting environments. Since too early anthesis and grain maturity may be highly related to the reduction of crop yield (Craufurd and Wheeler 2009; Olesen and Børgesen et al. 2012; Kristensen and Schelde et al. 2011; Patil and Laegdsmand et al. 2012), this scientific gap is important for the genetic improvement of crops to mitigate the risk of food shortage under global climate change.

Barley is one of the major annual cereals cultivated globally, and it is also an excellent model for investigating plants and agriculture in response to climate change (Dawson and Russell et al. 2015; Newton and Flavell et al. 2011). As the wild progenitor of cultivated barley (*H. vulgare ssp. vulgare*), wild barley (*Hordeum spontaneum* K. Koch) is believed to be an important resource for the genetic improvement of cultivated cereals under a changing climate (Nevo and Shewry 1992; Nevo and Zohary et al. 1979). By resurrecting ten wild-barley populations collected from Israel in 1980 (referred to as the ancestral populations in this study) and again the same populations in 2008 (referred to as the descendant populations in this study), in common garden investigations, Nevo and Fu et al. (2012) detected profound adaptive changes in the flowering times of all of these populations after 28 generations. In detail, they found that the average flowering time of all ten wild-barley populations collected in 2008 was significantly advanced by ~10 days, compared with the ancestral populations. Furthermore, simple sequence repeat (SSR) analysis showed that the allelic frequencies of SSR genotypes have been shifted in the descendant populations, strongly suggesting that after 28 generations, the genetic diversity in *H. spontaneum* was altered to adapt to global warming (Nevo and Fu et al. 2012).

However, investigations of neutral markers cannot address the mechanisms of adaptive evolution of early flowering. To investigate whether genes associated with regulation of flowering time also show evidence of selection in response to climate change, we chose the same material of wild barley as in Nevo et al.’s work (2012) as a model to reveal the potential signals of adaptive evolution under 28 years of global climate change. By contrasting the spatial and temporal patterns of variation in putative candidate flowering time genes, together with reference genes that have no function with the regulation of flowering time, we address the following objectives: (i) whether the evolutionary fates of alleles differed between flowering time candidate genes and reference genes; (ii) despite the environmental heterogeneity, whether all ten populations have undergone parallel changes. Specifically, we question whether the same genetic mechanisms are involved in adaptation to global warming across the ten populations (independent parallel evolution) or whether different molecular pathways result in a similar shift in phenotypes (convergent evolution)?

By focusing on genetic variations over both spatial and temporal scales, this study will highlight the genetic basis underlying the phenological shifts in flowering of wild barley adapting to environmental changes. It supplies a new example for understanding the molecular mechanism underlying the flowering time shifts in annual plants in response to environmental changes within a relatively few generations. This study would also provide important insights for genetic conservation in crop breeding and agricultural management in facing global climate change.

## Materials and methods

### Plant materials and DNA isolation

All of the plant materials used in this study were provided by Prof. Nevo, Institute of Evolution, University of Haifa, and all of the genotypes were matched to those sampled in the previous study (Nevo and Fu et al. 2012, Fig. 1). We planted these seeds in Shapotou Desert Research and Experiment Station from 2012 to 2014 and collected fresh leaves from a total of 275 individuals from both sample time points, with 4–8 individuals from each population (Supplementary Table S1). Fresh leaves were dried and preserved with silica gel, and all of the voucher specimens were deposited in the Key Laboratory of Stress Physiology and Ecology in Cold and Arid Regions, Department of Ecology and Agriculture Research, and Northwest Institute of Eco-Environment and Resources, Chinese Academy of Sciences. The total genomic DNA was extracted from the dried leaf tissue using a Plant Genomic DNA Kit (Tiangen Biotech Co., Ltd., Beijing, China) and was preserved at −80 °C.

**Fig. 1:**
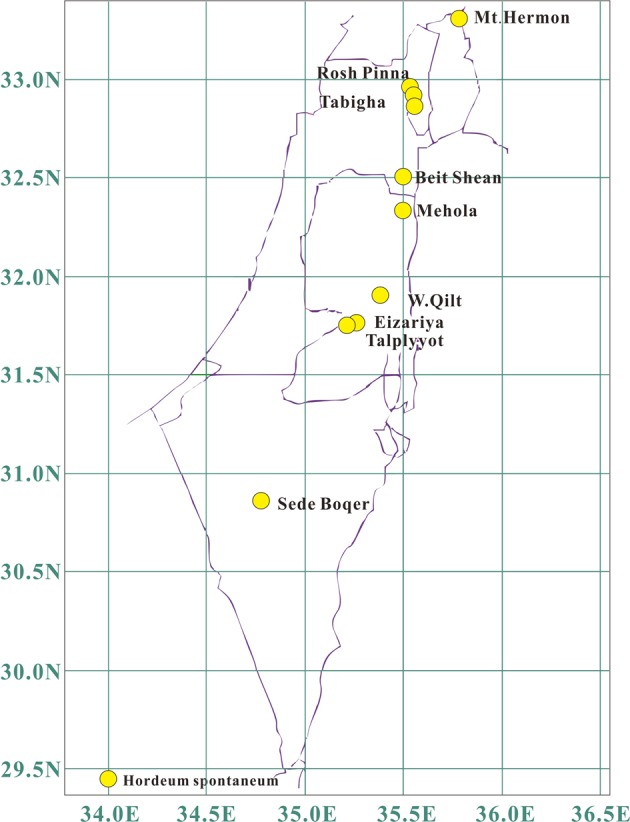
Distribution of the ten populations sampled in 1980 and again in 2008

### PCR amplification, sequencing, and sequence alignment

A total of 54 pairs of PCR primers (one pair for each gene) were designed by the PRIMER3 software (http://frodo.wi.mit.edu/primer3/) from putative flowering time genes that were either retrieved from those annotated in the National Center for Biotechnology Information databases (NCBI, http://www.ncbi.nlm.nih.gov/) or orthologs in barley identified from local BLAST alignments to the known flowering time genes from *A. thaliana* and rice (alignments with *e*-values < 10^−5^, Supplementary Table S2). For preliminary screening, each fragment was sequenced in four DNA pools, two ancestral and two descendant populations, each of which contained ten individuals. To ensure the equimolarity of the individuals in each pool, we measured and diluted DNA from all of the individuals to a concentration of 20 ng/μl in the four pools with a Nanodrop instrument. After the preliminary screening, based on their high number of polymorphic sites (Supplementary Table S3), we finally selected ten core genes (*FLOWERING TIME LOCUS T 1*, *FT1*; *FLOWERING TIME LOCUS T-LIKE PROTEIN 5*, *FT5*; *CONSTANS-LIKE PROTEIN 1*, *CO1*; *ZEITLUP*, *ZTL*; *PSEUDO-RESPONSE REGULATOR 7*, *Ppd-H1*(*PRR7*); *PHYTOCHROME B*, *PHYB*; *EARLY FLOWERING PROTEIN 3*, *ELF3*; *FLOWERING LOCUS C*, *FLC*; *VERNALIZATION-H2*, *VRN-H2*; *TIMING OF CAB EXPRESSION 1*, *TOC1* (Supplementary Table S2), and one promoter fragment of *FT1*, which are mostly conserved across seed plants, as the putative candidate genes for flowering time in wild barley. Similarly, we also chose nine unigene fragments from Bedada and Westerbergh et al. (2014) as the reference genes in this study, the functions of which have not been proved to be relevant to the flowering time (Supplementary Table S3).

All 20 final gene fragments were amplified in both the ancestral and descendant samples using 2× Taq Plus high-fidelity PCR MasterMix (Tiangen, Beijing, China) in a Gene-Amp PCR system 9700 DNA Thermal Cycler (PE Applied Biosystems, Norwalk, USA) with the programs listed in Supplementary Table S3. PCR products were purified with TIAN quick Midi Purification Kits (Tiangen, Beijing, China) and were Sanger-sequenced with both forward and reverse primers on an ABI 3130xl Genetic Analyzer (Applied Biosystems) with ABI Prism BigDye Terminator Cycle, version 3.1. DNA sequences were edited using BioEdit software, version 7.1.3 (Hall 1999), and then were aligned with MUSCLE (Edgar 2004). All heterozygous sites were visually checked in BioEdit and were separated by PHASE using DnaSP, version 5.10.01 (Librado and Rozas 2009; Stephens and Smith et al. 2001). The structures of all gene fragments were defined based on their best hits of BLAST on ESTs (Expressed Sequence tags) and genomic sequences of barley in NCBI, and new sequences were deposited in GenBank under accession numbers KX506100–KX506705.

### Data analysis

Genetic diversity was estimated for each gene fragment at the two time points by calculating the number of haplotypes (*H*), haplotype diversity (Hd), the number of segregating sites (*S*), and nucleotide diversity (*π* (Nei 1987); *Ө*_w_ (Watterson 1975)) for all sites, silent sites, and nonsynonymous sites. We also estimated the ratios of replacement and synonymous polymorphisms (*π*_a_/*π*_s_) for each fragment at each time point. All of the statistics were computed using DnaSP software, version 5.10.01 (Librado and Rozas 2009).

Previous research has reported a high level of ecogeographical population structure in barley across Israel (Bedada and Westerbergh et al. 2014; Morrell and Lundy et al. 2003; Nevo and Beiles et al. 1986), reflecting that the ecological background of these populations would affect the population structure of barley in this area. To investigate whether the population structure of these ten populations has changed through the 28 generations, we utilized the Bayesian genotypic clustering method InStruct (Gao et al. 2007) to delineate clusters of individuals based on multilocus single-nucleotide polymorphisms (SNPs) from candidate genes, reference genes, or the combined dataset across all populations in both ancestral and descendant samples. Polymorphic sites in strong linkage disequilibrium with other sites, as determined by a significant Fisher’s exact test after Bonferroni’s correction, were excluded from this analysis. To estimate the number of clusters, INSTRUCT was run for *K* = 2 to *K* = 10 in mode 2 for joint inference of population-selfing rate and population substructure for ten independent chains, each chain with 200,000 iteration steps, 100,000 burnins, and a thinning interval of ten steps, assuming different starting points. The graphical representations of population assignments from INSTRUCT were produced with DISTRUCT (Rosenberg 2002). Principal coordinate analysis (PCoA) was also used to estimate the genetic structure based on genetic distances. The genetic distance matrix used in PCoA was calculated using the FREQUENCY tool in GenAlex software, version 6.5, and then was visualized with the PCoA tool.

To detect any deviation in the site frequency spectrum caused by selection or population history, three neutrality tests were performed on the 20 gene fragments (11 putative candidate gene fragments and 9 reference gene fragments). These neutrality tests included Tajima’s *D* (Tajima 1989), the *DH* test (Zeng and Fu et al. 2006), and the MFDM (the maximum frequency of derived mutations) test (Li 2011). Tajima’s *D* is sensitive to low- and intermediate-frequency variants and was estimated with Arlequin, version 3.11 (Excoffier and Laval et al. 2005). As a compound test of Tajima’s *D* and Fay and Wu’s *H* (Fay and Wu 2000), the *DH* test is useful for detecting the potential signal of directional selection. Based on the maximum frequency of derived mutations, the MFDM test is a summary statistic calculated from a coalescent tree topology and is known to be robust to the confounding impacts of population structure and history events. The latter two tests were performed using *Aegilops tauschii* as an outgroup (Jia and Zhao et al. 2013).

To further distinguish whether signals obtained from different SNPs were the results of global warming or environmental heterogeneity, two methods were used to screen the SNPs putatively involved in local adaptation at the two time points. All of the indels were encoded as “pseudo-SNPs” for further analysis. First, to identify SNPs under spatial-diversifying selection, we used an *F*_ST_-based outlier approach based on the multinomial-Dirichlet model implemented in BayeScan, version 2.1 (Foll and Gaggiotti 2008). Compared with neutral SNPs, SNPs under diversifying selection should have higher values of *F*_ST_, while the balancing selection should result in lower *F*_ST_ values (Beaumont and Nichols 1996; Foll and Gaggiotti 2008). Second, we also used the BAYENV2 program (Günther and Coop 2013) in both ancestral and descendant populations to test for correlations between the differentiation of the allele frequencies and two environmental variables (mean annual temperature and mean annual rainfall). The program implemented a Bayesian generalized linear mixed model that assumes that the allele frequencies of all populations are correlated due to a shared ancestry (mean of multivariate normal distribution), and it also allows for variation caused by random drift (a correlation–covariance matrix). The environmental variable is introduced in an alternative model with a linear effect, and the significance is evaluated by a Bayes factor computed from a comparison of the likelihoods of the alternative model to the null model. After excluding sites that showed significant linkage or that had missing alleles, all of the SNPs from ancestral and descendant datasets were used to estimate the correlation–covariance matrix. The covariance matrices with a 1,000,000-step Monte carlo Markov Chain (MCMC) were used in the subsequent tests of the correlation of environment variables to allele frequency differences in all of the SNPs from ancestral and descendant populations, with ten runs of 1,000,000 MCMC steps with different random seeds, respectively. The top 5% Bayes factors (BFs) of the averaged results across the ten runs combined with high values of Spearman’s *ρ* were then considered as robust candidates, as suggested by the manual.

Finally, temporal selection indices (*s*) were estimated for 392 SNPs, excluding triallelic SNPs or SNPs specific to the descendant populations using a Wright–Fisher ABC-based approach (WFABC (Foll and Shim et al. 2015)), which estimates the parameters of a Wright–Fisher model with selection by allele frequencies sampled at different time points. First, the effective population size (*N*_e_) at every locus was estimated by an approximate Bayesian computation approach, and then simulations of *s* for each locus were performed by a Wright–Fisher model with the initial allele frequency and estimated *N*_e_ from the first step. Since directional selection would greatly shift the allele frequencies between the ancestral and descendant populations, in this study, SNPs/indels with high values of *s* and high allele frequencies in each descendant population were regarded as target SNPs that were subjected to directional selection due to global warming.

On the other hand, to further analyze the effects of genetic drift on allele frequency change over the past 28 generations, the evolutionary fates of the SNPs/indels were also simulated with PopG genetic simulation program, version 4.03 (http://evolution.gs.washington.edu/popgen/), with a start allele frequency of 0.9 and *N*_e_ of 1000 for ten populations.

As a highly selfing plant (Brown and Zohary et al. 1978; Lin and Morrell et al. 2002), linkage disequilibrium (LD) should be common between SNPs and indels from the same network regulating flowering time in wild barley due to hitchhiking. Thus, to further verify the coadaptation of the target SNPs/indels, we compared patterns of LD between all SNPs/indels at the two time points, and between the top target SNPs/indels by Haploview (Barrett and Fry et al. 2005). According to the manual, LD was characterized by calculation of the squared correlation coefficient *r*^2^, a conventional measure of association that is equal to the square of the correlation coefficient between the two alleles (Weir 1996).

## Results

### Patterns of nucleotide diversity in the ancestral and descendant populations

A total of 8614-bp SNPs were sequenced for the 11 candidate fragments, with length ranging from 606 to 1564 bp and an average length of 783 bp (Supplementary Table S3). A total of 270 SNPs were identified in the materials sampled in 2008, with 63 located in coding regions. The nucleotide diversity per site (*π*) ranged from 7.3 × 10^−4^ to 1.6 × 10^−2^, and diversity based on the number of segregating sites (*Ө*_w_) ranged from 1.4 × 10^−3^ to 2.1 × 10^−2^ (Supplementary Table S4). In the ancestral populations, 255 SNPs were identified, with 54 located in coding regions (Supplementary Table S3) and with values of *π* and *Ө*_w_ ranging from 5.9 × 10^−4^ to 1.3 × 10^−2^ and from 2.0 × 10^−3^ to 1.9 × 10^−2^, respectively (Supplementary Table S4). Compared with the descendant populations, all of the diversity parameters in the ancestral populations were slightly lower (*p*-value > 0.05, Supplementary Fig. S1), with the exception of silent sites. In both the ancestral and descendant populations, all of the values of *π*_a_/*π*_s_ at candidate gene fragments were <1 except at *CO1*, which harbored a *π*_a_/*π*_s_ over 1 in the descendant populations (Supplementary Table S4).

The total length of the nine reference gene fragments sequenced was 7133 bp (ranging from 656 to 950 bp for each gene fragment, Supplementary Table S3). Unlike the higher level of variation found in candidate genes in the descendant populations, the average nucleotide diversity for the reference gene fragments was slightly greater in the ancestral populations (*p*-values > 0.05, Supplementary Fig. S1), with *π* ranging from 1.9 × 10^−4^ to 1.1 × 10^−2^ in the ancestral populations and from 4.3 × 10^−4^ to 1.2 × 10^−2^ in the descendant populations. All of the values of *π*_a_/*π*_s_ at these loci were <1 at both time points, except for *UG20519* in the ancestral populations (Supplementary Table S4).

Neutrality tests showed that in the ancestral populations, only one reference gene (*UG11973*) appeared to be under positive selection by all three methods (Table 1), while none of the candidate genes showed significant deviations from neutrality. In the descendant generation, however, two candidate genes (*CO1* and *ZTL*) and one reference gene (*UG22211*) showed significant signs of positive selection in all three neutrality tests (Table 1).

**Table 1:** Neutrality tests for 11 putative candidate genes and nine neutrality reference genes

Locus	Tajima’s *D*		*p*-value of MFDM		*p*-value of the *DH* test	
	2008	1980	2008	1980	2008	1980
Flowering time genes						
*FT1* (*p*)	0.510	0.738	0.729	0.602	0.563	0.574
*FT1* (*CDS*)	0.844	0.343	0.112	>0.005	0.835	0.559
*FT5*	−0.710	−0.368	>0.010	0.070	0.106	0.248
*ELF3*	−1.651 (0.020)	−1.207	>0.017	>0.013	0.049	0.031
*TOC1*	−0.991	−0.851	>0.025	0.195	0.041	0.063
*ppD-H1*	−0.754	−0.775	>0.010	>0.013	0.121	0.101
*CO1*	−1.703 (0.018)	−1.482 (0.026)	0.016	1	0.004	0.177
*ZTL*	−1.779 (0.008)	−1.287	0.015	0.091	0.004	0.033
*phyB*	−0.172	−1.240	1	1	0.744	0.288
*VRN-H2*	−0.628	−0.882	>0.003	>0.004	0.143	0.123
*FLC*	−1.416 (0.041)	−1.545 (0.024)	1	1	0.375	0.340
Reference gene						
*UG11973*	−1.155	−1.332 (0.044)	0.039	0.042	0.015	0.005
*UG13006*	−0.152	0.657	>0.004	>0.007	0.345	0.703
*UG14513*	−1.411 (0.042)	−1.352	n.a.	n.a.	n.a.	n.a.
*UG20519*	1.815	2.242	>0.01	>0.01	0.935	0.971
*UG22211*	−1.582 (0.021)	−1.469 (0.035)	0.015	>0.013	0.012	0.022
*UG23391*	−1.153	−0.449	0.096	>0.013	0.032	0.199
*UG23683*	−0.978	−0.409	>0.017	0.043	0.186	0.491
*UG27456*	0.148	−0.863	n.a.	n.a.	n.a.	n.a.
*UG32388*	−0.789	−1.254	0.016	0.015	0.087	0.028

### Temporal changes in population differentiation and spatial-diversifying selection

**Fig. 2:**
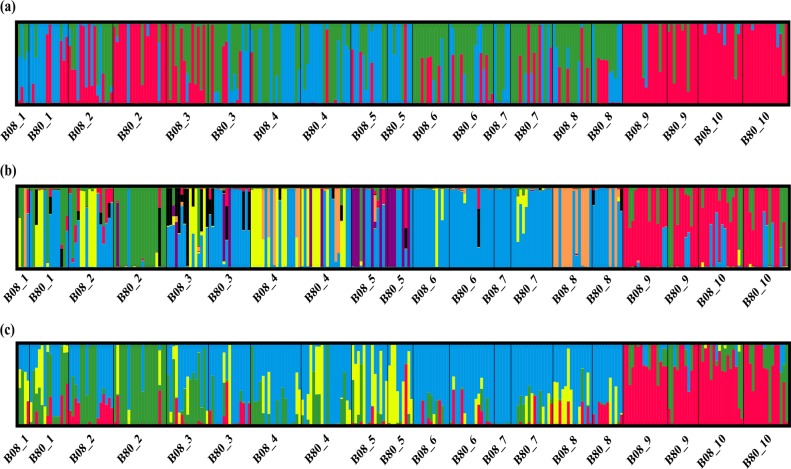
Population structure in ten wild-barley populations inferred with INSTRUCT based on SNPs/indels of reference genes, candidate genes, and concatenated genes of both reference genes and candidate genes, respectively. **a** Population structure based on SNPs/indels of reference genes. **b** Population structure based on SNPs/indels of candidate genes. **c** Population structure based on SNPs/indels of concatenated genes

INSTRUCT analysis predicated *K* = 3, *K* = 7, and *K* = 4 as the optimum population structure for reference genes, candidate genes, and the total dataset, respectively. Compared with the genetic components of the neutral dataset of reference genes, the genetic components of candidate genes were quite different between the two time points, and in descendant populations, different genetic components were detected in descendant populations 4, 5, and 8 compared with those ancestral populations (Fig. 2). Furthermore, the genetic components of most populations had also shifted during the last three decades in all the three datasets, and the greatest shifting was detected in the candidate flowering time genes for population 2. These results were also supported by PCoA analysis (Supplementary Fig. S2). Moreover, BayeScan results showed that in the ancestral populations, the average values of *F*_ST_ were 0.382 and 0.381 for candidate genes and reference genes, respectively, while in the descendant populations, the average values of *F*_ST_ were 0.469 and 0.462 for candidate genes and reference genes, respectively (Fig. 2b). Based on the *t*-test, the significantly higher average values of *F*_ST_ for both candidate loci and reference loci in the descendant populations than in the ancestral populations suggest that genetic differentiation had been significantly enhanced over the past 28 generations.

**Fig. 3:**
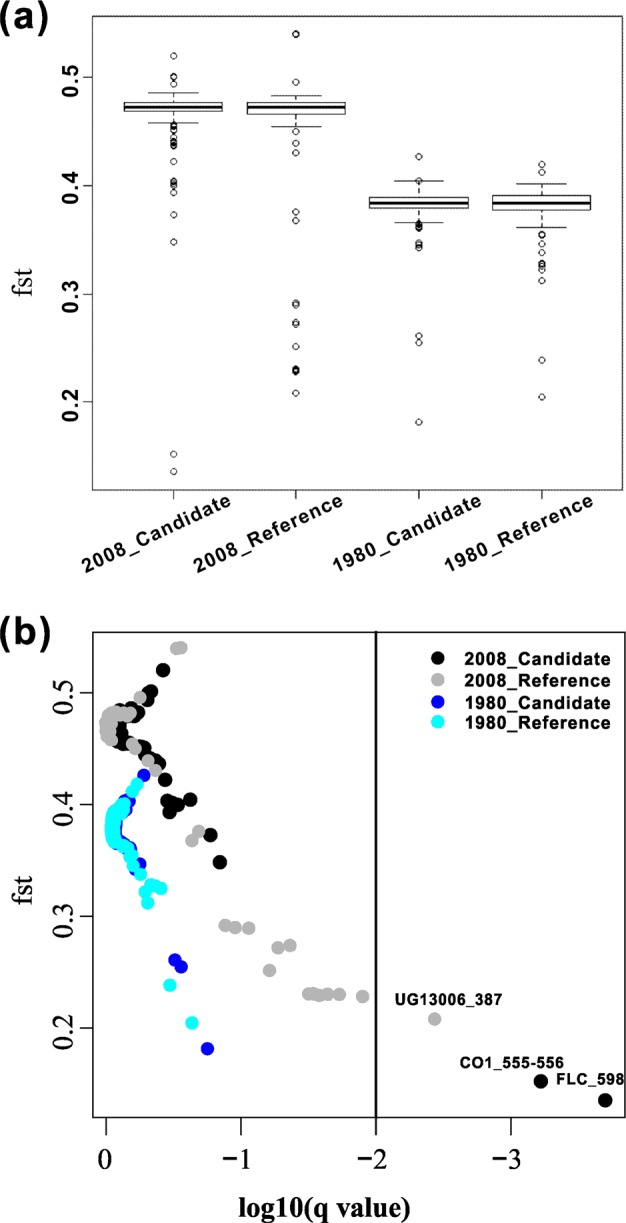
Results of BayeScan. **a** Boxplots of *F*_ST_ values based on candidate and reference loci in two-generation populations, respectively. **b** *F*_ST_ outliers of both ancestral and descendant populations. The *x-*axis represents the *q-*value, which standardized with log10, the *y*-axis represents the value of *F*_ST_. Plots with black color represent the candidate SNPs/indels in the ancestral populations, gray color represents the reference SNPs/indels in the descendent populations, while blue and light blue represent the candidate SNPs/indels and reference SNPs/indels in the ancestral populations, respectively

As shown in the *F*_ST_ outlier analysis (Fig. 3), no SNP/indel was found to be under diversifying selection in either ancestral or descendant populations, whereas three SNPs/indels (*CO1*_555–556, *FLC*_598, and *UG13006*_387) were suggested to be under balancing selection in the descendant populations as their *F*_ST_ values were lower than the average *F*_ST_ values.

The BAYENV analyses showed that most of the SNPs/indels that were found in the top 5% of BFs and with absolute values of Spearman’s *ρ* > 0.5 were found in the reference genes. In particular, the allele frequencies at *UG32388*_464 were strongly associated with heterogeneity of the mean annual temperature at both time points (BF = 7.74, *ρ* = −0.60 in ancestral populations, BF = 11.19, *ρ* = −0.67 in descendant populations, Table 2).

**Table 2:** Details of the top 10% SNPs/indels that correlated with mean annual temperature and mean annual rainfall

Mean annual temperature					Mean annual rainfall				
Rank	SNPs/indels	Bayes factors	Spearman’s *ρ*	Annotations	Ranks	SNPs/indels	Bayes factors	Spearman’s *ρ*	Annotations
Ancient populations sampled in 1980									
1	*UG32388*_464	7.74	−0.60	T/C (SS)	1	*ELF3*_89-92	3.22	−0.22	TCTT (±) (Int.)
2	*UG27456*_409	4.21	−0.56	A/G (Int.)	2	*VrnH2*_247	3.21	0.13	C/G (SS)
3	*FT1C*_1477	3.12	0.65	C/T (SS)	3	*UG27456*_414	3.07	0.22	C/T (Int.)
4	*ELF3*_89-92	2.90	0.26	TCTT (CT) (Int.)	4	*CO1*_596	2.09	−0.16	A (±) (Int.)
5	*UG27456*_414	2.86	−0.26	C/T (Int.)	5	*UG22211*_10	209	−0.16	A/G (Int.)
6	*ELF3*_219	2.47	−0.31	C/G (Int.)	6	*UG23391*_804	2.09	−0.16	G/C (Int.)
7	*FT5*_214	2.29	0.37	G/A (Int.)	7	*ELF3*_209	2.04	0.16	G (±) (Int.)
8	*UG20519*_166	2.10	−0.32	C/T (Int.)	8	*FT1C*_1369-1370	2.04	0.16	TG (±) (Int.)
9	*UG20519*_186	2.08	−0.51	T/C (Int.)	9	*UG32388*_464	2.03	0.31	T/C (SS)
Descendant population samples in 2008									
1	*UG32388*_464	11.19	−0.67	T/C (SS)	1	*UG22211*_179	13.10	−0.29	A/G (SS)
2	*UG22211*_179	10.54	0.35	A/G (SS)	2	*phyB*_103	5.76	−0.18	A/G (SS)
3	*UG23391*_791	6.62	−0.41	C/T (Int.)	3	*ELF3*_9	4.62	0.19	C/T (SS)
4	*VrnH2*_228	4.12	−0.19	T/A (SS)	4	*FT1P*_25	4.62	0.19	G/A (5ʹUTR)
5	*UG23683*_294	4.12	−0.19	G/A (Int.)	5	*UG13006*_1	4.62	0.19	T/C (Int.)
6	*ELF3*_9	4.08	−0.25	C/T (SS)	6	*UG13006*_603	4.62	0.19	G/T (Int.)
7	*FT1P*_25	4.08	−0.25	G/A (5ʹUTR)	7	*UG20519*_468	4.62	0.19	G/A (Int.)
8	*UG13006*_1	4.08	−0.25	T/C (Int.)	8	*FT1P*_18	4.62	−0.19	T/C (5ʹUTR)
9	*UG13006*_603	4.08	−0.25	G/T (Int.)	9	*FT5*_278	4.62	−0.19	A/G (Int.)
10	*UG20519*_468	4.08	−0.25	G/A (Int.)	10	*UG13006*_120	4.62	−0.19	A/G (Int.)
11	*FT1P*_18	4.03	0.25	T/C (5ʹUTR)	11	*UG13006*_434	4.62	−0.19	G/C (Int.)

### Directional selection over the past three decades

**Fig. 4:**
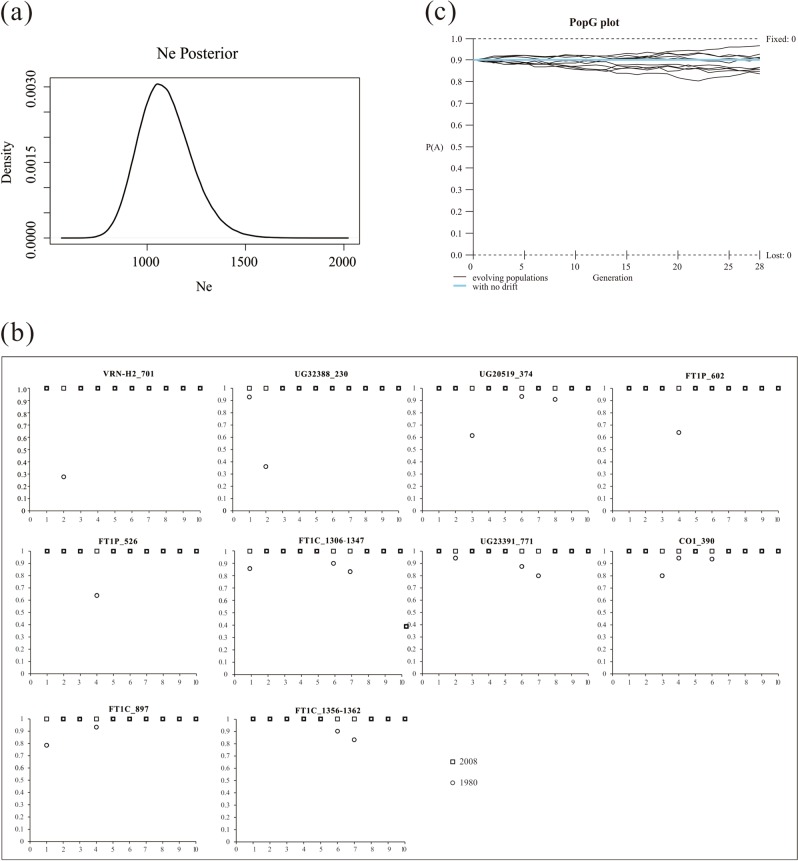
**a** Effective population size (Ne) of wild barley performed with WFABC software based on SNPs/indels from ten wild-barley populations sampled in 1980 and again in 2008. The *x-*axis represents the posterior for Ne, the *y-*axis represents the distribution density for Ne. **b** Plots of allele frequency for the top ten SNPs/indels under strong selection. Plots with light-blue color represent loci from the ancestral populations, while red color represents loci from the descendant populations. The *x*-axis represents the populations, while the *y*-axis represents the allele frequencies in populations. **c** Simulation of the effect of genetic drift on the allele frequency fixed in the descendant populations with the PopG software. The initial *A* allele frequency, *P*(*A*) was set to 0.9, the *x-*axis represents the generations, the number of evolving populations was 10, and no selection acts on them

The results of WFABC showed that the average value of *N*_e_ was >1000 (Fig. 4a), and the mean selection index *s* over the last 28 generations was significantly lower in the reference SNPs/indels than in the candidate genes (*p*-value > 0.05, Fig. S3). Based on the positive relationships between the allele frequency changes and selection indices among the SNPs/indels fixed in the descendant populations (Supplementary Fig. S4), we found a total of 54 SNPs/indels (26 SNPs in candidate genes vs. 28 SNPs in reference genes) out of 392 SNPs/indels that were under strong selection with values of *s* > 0.4. To reduce the false-positive rate, we selected only the top ten SNPs/indels (including seven candidate and three reference genes) for further analysis (Table 3 and Fig. 4b). As illustrated in Fig. 4b, all of these SNPs/indels were fixed in the descendant populations, whereas seven of them were polymorphic in at least two ancestral populations. Nevertheless, the results of PopG simulations strongly suggested that given the estimated effective population sizes, no SNPs should be fixed by a drift over such a short time period (Fig. 4c).

**Table 3:** Details of the top ten SNPs/indels with strong selection

Rank	SNPs/indels	Selection index	Annotations
1	*VRN-H2*_701	−0.610	C/G (Int.)
2	*UG32388*_230	−0.602	C/G (Int.)
3	*UG20519*_374	0.578	T/G (Int.)
4	*FT1P*_602	0.572	A/G (5ʹUTR)
5	*FT1P*_526	−0.571	G/A (5ʹUTR)
6	*FT1C*_1306-1347	−0.569	TAGGTACGTATATCCCGGGTGTACGTACATGTGTGTAGCTCA(±) (Int.)
7	*UG23391*_771	0.567	G/C (Int.)
8	*CO1_*390	−0.551	A/G (NSS:Val/Ile)
9	*FT1C*_897	0.545	T/C (Int.)
10	*FT1C_*1356-1362	−0.541	TAGCTCA (±) (Int.)

Interestingly, five of the top ten SNPs/indels came from the *FT1* gene, with two located in the promoter region and three in introns. We found that all of the remaining SNPs, with the exception of the nonsynonymous mutation *CO1*_390, were located in noncoding regions, suggesting that they might affect the pre- and/or post-transcriptional regulation of *FT1*.

The result of Haploview showed that the top ten SNPs/indels with strong selection were not linked to each other in the ancestral populations, except for two SNPs from the *FT1* gene: *FT1P*_526 and *FT1P*_602. Moreover, among the 54 SNPs/indels under strong selection, we found that 13 pairs of SNPs/indels were in strong linkage disequilibrium in the ancestral populations (*r*^2^ > 0.8), with 11 of them located in the same genes (Supplementary Table S8).

## Discussion

In this study, based on the same ten populations sampled at two time points, we investigated the evolutionary fates of the candidate and reference SNPs/indels in samples from the same wild-barley populations sampled in 1980 and 2008 (Nevo et al. 2012), and attempted to determine how local adaptation and parallel evolution have influenced the fates of allelic variations in response to global climate change.

### Few local adaptation signals detected among Israeli wild-barley populations at two time points

Previous investigations have documented a strong population differentiation based on several neutral nuclear genes in wild barley (Bedada and Westerbergh et al. 2014; Morrell and Lundy et al. 2003; Nevo and Beiles et al. 1986). Here, we found a stronger population differentiation in the descendant populations than in the ancestral populations based on both candidate genes and reference genes (Fig. 3b), indicating that wild barley in Israel has highly diverged as a result of the ecogeographical adaptation (Bedada and Westerbergh et al. 2014; Morrell and Lundy et al. 2003; Nevo and Beiles et al. 1986; Nevo et al. 2012). However, to our surprise, based on the *F*_ST_-based outlier approach, no SNP/indel was found to be under diversifying selection to adapt to the heterogeneous local environments, since a high level of ecogeographical population structure was found in barley across Israeli natural populations investigated. Only three outlier SNPs/indels with low values of *F*_ST_ were found, which suggested to be under a balancing selection (Beaumont and Nichols 1996; Foll and Gaggiotti 2008).

Basically, the high values of *F*_ST_ among wild-barley populations resulting from environmental heterogeneity would provide a great chance to detect signals of local adaptation among these populations. However, in this study, we failed to detect any strong signal of local adaptation based on high population differentiation for either the flowering time genes or the unannotated reference genes, which suggested that the drift could have increased the genetic structure of wild barley during the last 28 generations. We also realized that the power to detect outlier loci with significantly greater genetic differentiation compared with the genomic background is much higher in plant species with a lower *F*_ST_, such as the outcrossing, wind-pollinated *Populus tremula*, *Picea abies*, and *Populus balsamifera* L. (Chen and Källman et al. 2016; Keller and Levsen et al. 2012; Ma and Hall et al. 2010). In this study, compared with these outcrossing plant species mentioned above, we found that the average *F*_ST_ of the genomic background was higher in wild barley, which would make it more difficult to detect significant outliers under spatial-diversifying selection from a statistical point of view. In contrast, it is relatively easier to identify outliers showing reduced differentiation in populations with a high level of *F*_ST_, and we indeed detected three such loci in the ten barley populations. Actually, the shortcomings of the *F*_ST_ outlier approach have been reported in other studies because the method relies on a very specific model of population structure, and violations of these assumptions affect the power to detect outliers (Duforet-Frebourg and Bazin et al. 2014). Furthermore, environmental association analyses performed with BAYENV identified only one SNP in the reference gene sets that was associated with the variation in the annual mean temperature among the populations. Since a high population structure negatively affects the power to detect signals of local adaptation, it seems that this currently popular method suffers from practical limitations in species such as wild barley.

### Adaptive signals of advanced flowering time of the ten wild-barley populations in Israel after 28 generations

Common garden investigations have shown that all the ten descendant populations flower at least 10 days earlier than the ancestral populations, regardless of the heterogeneity of the environment (Nevo and Fu et al. 2012). We thus proposed that the local environment might contribute to the divergence of flowering time among populations, but has little influence on the early flowering phenotype in all the ten descendant populations of wild barley. Early flowering has also been found in numerous wild plants across many different environments in response to the global climate change (Bock and Sparks et al. 2014; Brunet and Larson-Rabin 2012; Craufurd and Wheeler 2009; Franks and Sim et al. 2007; Hovenden and Williams et al. 2008; Miller-Rushing and Primack 2008). Therefore, early flowering observed in all the ten descendant populations of wild barley is likely not related to small-scale variations among local environments but rather to the overall global climate change, as an effective strategy in facing the long-term trends of warmer climates (Munguía-Rosas et al. 2011).

To fully understand the effects of global warming on the early flowering phenology in wild barley, based on temporal changes in allele frequencies, we estimated the strength of selection acting on standing genetic variations over the intervening 28 years. To our surprise, we found that 54 out of 392 SNPs/indels (~13.8% of all the standing variation) could be under a strong directional selection (*s* > 0.4, Supplementary Table S7). For example, one of them (*CO1*_390, Table 3) came from *CO1*, which was also detected to be significantly under positive selection in the descendant populations with multiple neutrality tests, including Tajima’s *D*, the *DH* test, the MFDM test, and *π*_a_/*π*_s_ (Table 1). Of course, we should be cautious in explaining the results of WFABC, because this software has not been used in plants yet, but only in a few viruses (Foll and Poh et al. 2014; Shim and Laurent et al. 2016). However, we noted that all of the SNPs/indels that deemed to be under strong selection were fixed in the descendant populations, while the initial frequencies varied among the different ancestral populations (Fig. 4b). Notably, genetic drift could enhance the genetic divergence between populations (Maruyama and Fuerst 1985), which we indeed witnessed in the descendant populations. Theoretically, genetic drift could also play a role in the fixation of the alleles during the last three decades. To our surprise, the results of PopG did not show any case results from genetic drift with 1000 times simulations in ten independent populations with a start frequency of 0.9. These data strongly supported the hypothesis that quick fixation of alleles in all the ten descendant populations could not result from genetic drift but rather by adaptive evolution. On the other hand, linkage disequilibrium among SNPs could facilitate the fixation of the alleles due to the hitchhiking effects (Hartfield and Otto 2011). Actually, we found only two out of 13 pairs of SNPs strongly linked to each other in the ancestral populations, which came from different genes (Supplementary Table S8). Considering the low-linkage disequilibrium of wild barley (Morrell et al. 2003), the hitchhiking effects thus could be ruled out in affecting the fixations of intergenic SNPs in the descendant populations. We thus propose that their fixations were mostly due to trans-regulation between genes from different chromosomes, which played coadaptation roles in mitigating the effects of rapid global climate change.

Notably, seven out of the top ten SNPs/indels under strong selection were from three downstream flowering time genes: *CO1*, *VRN-H2*, and *FT1*. *CO1* is the closest barley ortholog of the *Arabidopsis CONSTANS* (*CO*) gene, which is a key photoperiod response gene that acts by promoting the transcription of *FT1*, which in turn promotes flowering (Campoli and Drosse et al. 2012; Griffiths and Dunford et al. 2003). *FT1* is believed to be the key regulator of flowering, and it plays an important role in integrating flowering signals from multiple pathways and then triggering the developmental switch, turning the shoot apex from vegetative to reproductive growth in barley (Faure and Higgins et al. 2007). In winter barley, the vernalization gene *VRN-H2* has been documented to be the major suppressor of flowering under long-day conditioning without vernalization. *VRN-H2* acts by downregulating the expression of the key floral integrator *FT1* on long days, while its activity was suppressed under short-day conditions (Dubcovsky and Chen et al. 2005; Trevaskis and Hemming et al. 2007; Yan and Loukoianov et al. 2004). Interestingly, recent research on the relationships of photoperiod and vernalization pathway genes in barley suggested that both the repressor *VRN-H2* and the integrator *FT1* were regulated by *CO1* (Mulki and von Korff 2016). Altogether with our investigation on spatial and temporal nucleotide diversity in the wild-barley populations, we propose that these three downstream flowering time genes played a critical role in wild-barley adaptation to global warming. To better understand the allelic effects of these strongly selected SNPs/indels and the precise regulation of these genes in promoting early flowering, we should focus on association studies with larger populations and functional analyses in the future. Such studies could help us to construct a more comprehensive flowering time network, yielding a more detailed picture of the genetic mechanisms underlying early flowering of wild barley in response to global climate change.

Based on temporal analysis of allele frequency shifts by WFABC, we found that five out of the top ten SNPs/indels under strong selection were located in the *FT1* gene (two SNPs were located in the promoter region, while the other three were located in the intron regions), and they were all fixed in the descendant populations. Previous studies have identified a variation in the promoter (Casas and Djemel et al. 2011), the first intron (Yan and Fu et al. 2006), and even the copy number of the *FT1* gene (Nitcher and Distelfeld et al. 2013), all of which have been associated with shifts in flowering time in barley. One such variant (*TC* vs. *AG* alleles), located in the first intron, was shown to be related to early flowering (Casas and Djemel et al. 2011; Yan and Fu et al. 2006), and it was also found to be segregated in wild-barley populations. Although neither of these two alleles was found to be fixed in the descendant populations of wild barley in this study, a new SNP in the promoter region of *FT1* (*FT1P*_526_(*G*/*A*)) under strong selection potentially affected the binding of transcription factors of the TALE homeodomain family of proteins, which have been suggested to be involved in developmental processes throughout the plant lifecycle (Hamant and Pautot 2010). As all of the individuals in the descendant populations harbored the *G* allele, which failed to form the TF motif TGACA (the resulting motif was TGGCA), we can speculate that the fixation of this *G* allele in the descendant populations could affect the regulation of the developmental processes that orchestrated early flowering in response to global climate change. Of course, more mutagenesis work on the promoter of *FT1* in barley could help us to test this hypothesis.

## Conclusions and prospects

The observation of advanced flowering in ten populations of wild barley after 28 generations is hypothesized to be an adaptive consequence to avoid the increased stress of environment changes, such as global warming. In this study, by thoroughly investigating the evolutionary fates of allelic variations on flowering time genes over three decades of environment changes, we found that genetic differentiation was significantly increased among the descendant populations, but had negligible effects on their local adaptation. Based on temporal analysis of allele frequency changes, we identified multiple unlinked SNPs/indels that were under strong directional selection, especially those from key components of flowering time regulation (*CO1*, *VRN-H2*, and *FT1*), which were fixed in all of the descendant populations and would together orchestrate early flowering to adapt to environment changes. This endeavor provides a novel example of understanding the genetic basis of parallel evolution in natural populations, and it could provide instructive genetic information for crop improvement in face of the rapidly growing world population and continuous global climate changes.

### Data archiving

All the new sequences can be archived in GenBank with accession numbers of KX506100–KX506705.

## Supplementary information

supplemental materials

supplemental materials
