# Supplementary material for: Adaptive signals of flowering time pathways in wild barley from Israel over 28 generations
Source: Heredity (Edinb). 2019 Sep 16;124(1):62–76. doi: 10.1038/s41437-019-0264-5 (PMC6906298; doi:10.1038/s41437-019-0264-5)
Supplement: Supplementary file 1 — supplemental materials [file 41437_2019_264_MOESM1_ESM.docx]

Table S1 A list of samples used in this paper and basic climatic characteristics of the 10 populations.

| **Population** | **Mean annual**  **rainfall (mm)** | **Mean annual temp. (℃)** | **Number of samples in 2008** | **Number of samples in1980** |
| --- | --- | --- | --- | --- |
| **1.Mt. Hermon** | **1400** | **11** | **4** | **14** |
| **2. Rosh Pinna** | **697** | **19** | **16** | **19** |
| **3. Tabigha, terra rossa** | **436** | **24.1** | **15** | **15** |
| **4. Tabigha, basalt** | **436** | **24.1** | **18** | **18** |
| **5. Bet Shean** | **290** | **22.8** | **13** | **9** |
| **6. Mehola** | **290** | **23** | **13** | **16** |
| **7. Wadi Qilt** | **144** | **24.8** | **6** | **15** |
| **8. Eizariya** | **380** | **20** | **14** | **11** |
| **9. Talpiyyot** | **486** | **18.2** | **16** | **11** |
| **10. Sede Boqer** | **91** | **19.4** | **16** | **16** |
| **Total** |  |  | **131** | **144** |


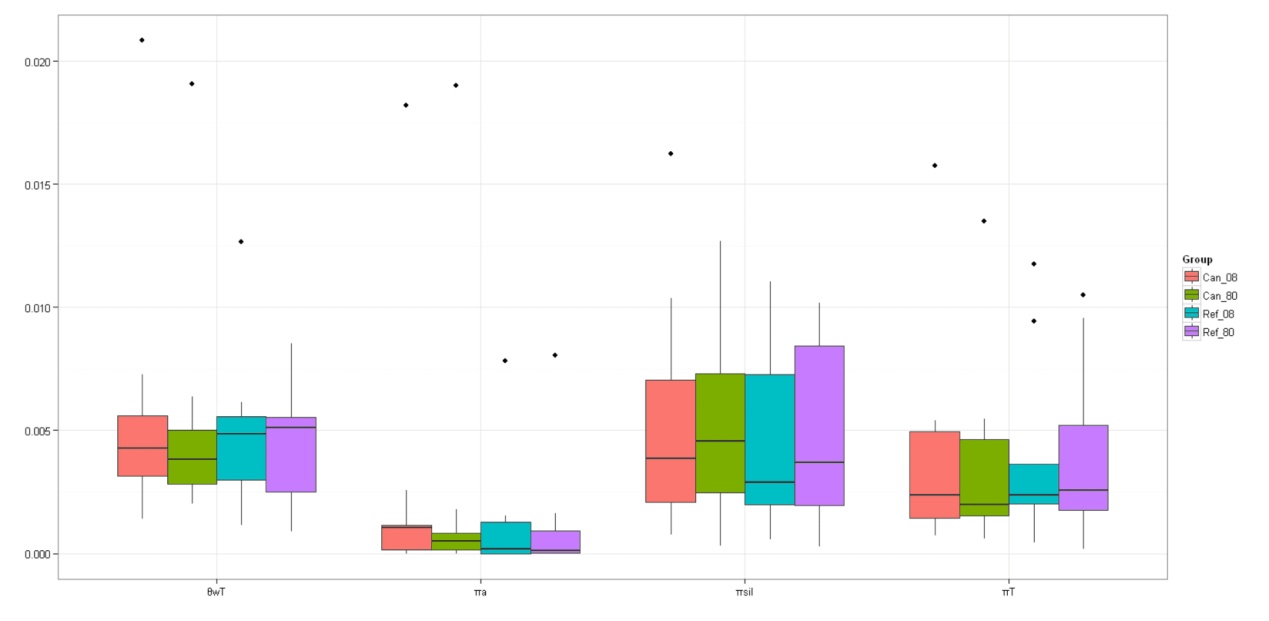


Fig. S1 Boxplot of polymorphisms for both candidate genes and reference genes at different time points.


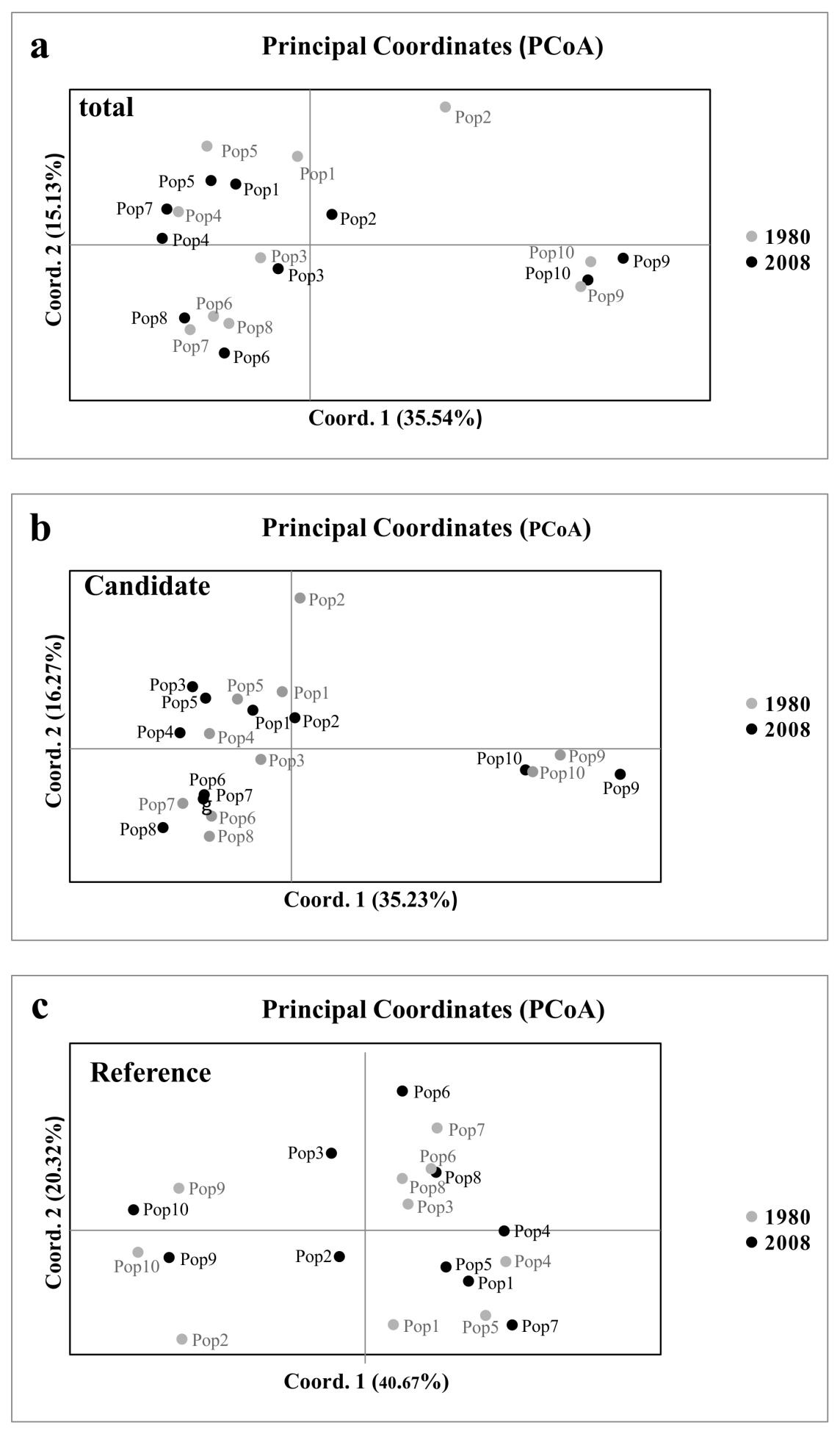


Fig. S2 The result of PCoA based on different gene fragments at two time points.


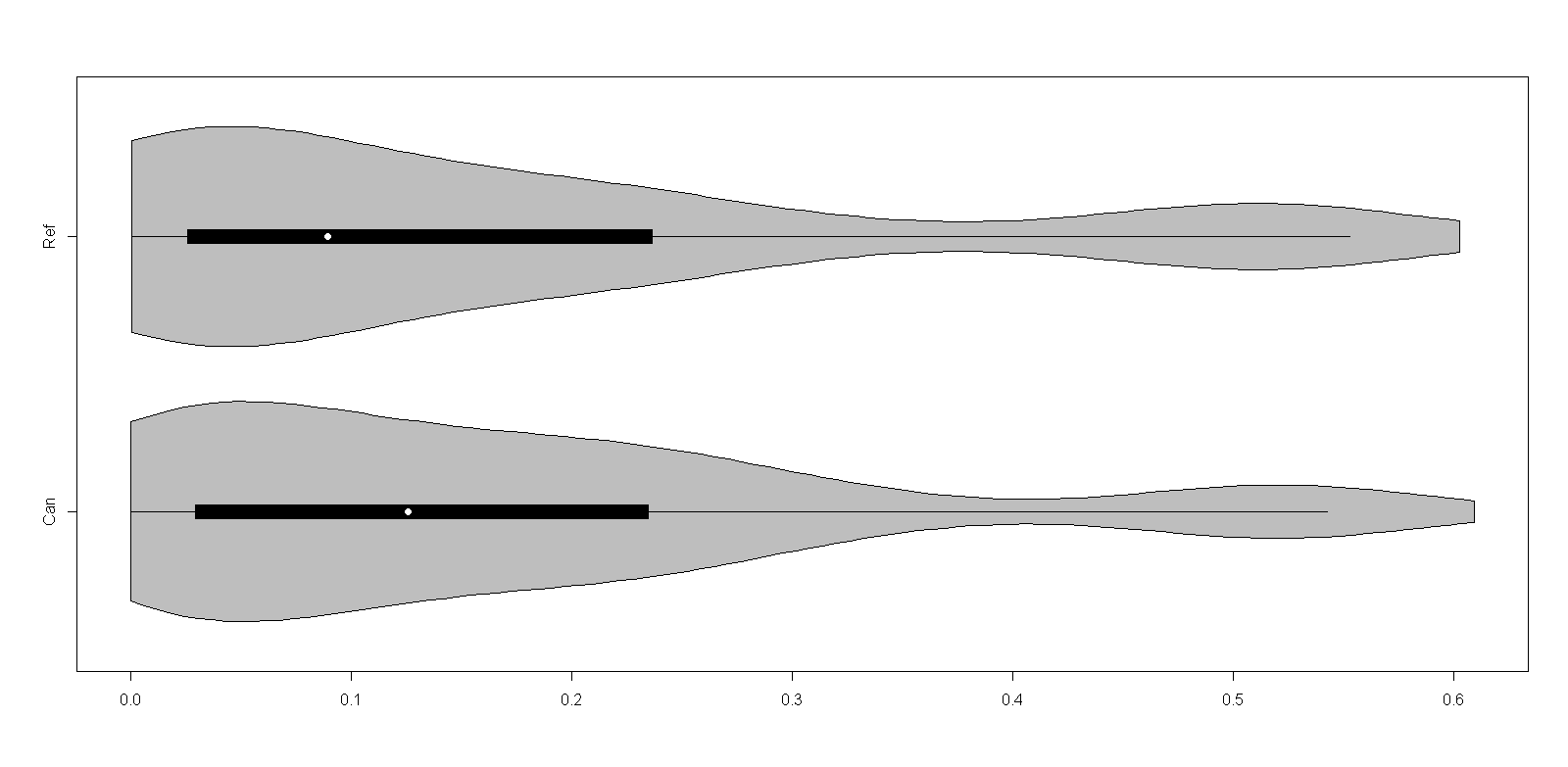


Fig. S3 Vioplots of selection indices for candidate loci and reference loci through 28 generations. Can represents candidate loci; Ref represents reference loci. The values used here of each SNP/indel were the absolute values.


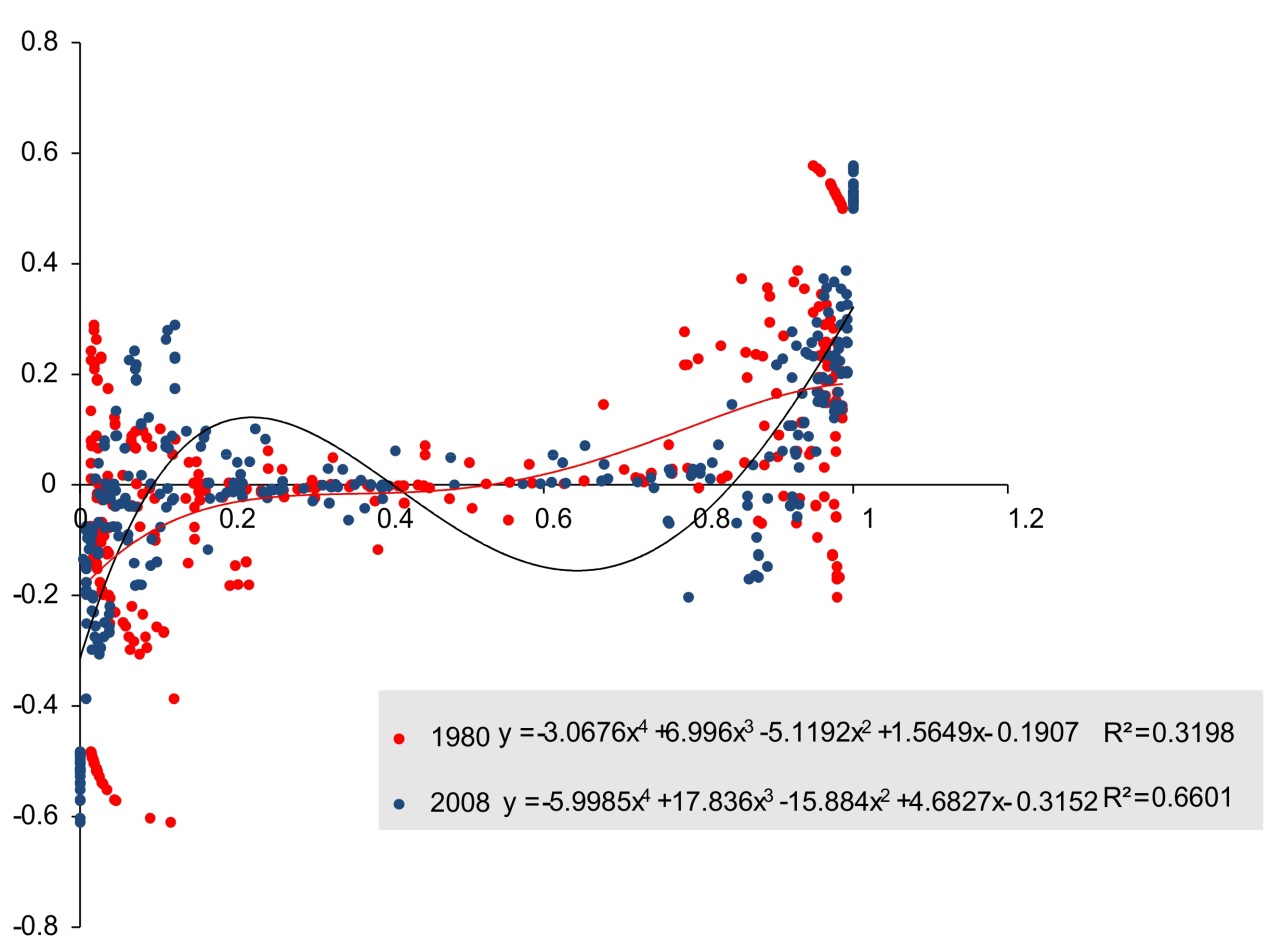


Fig. S4 Plot of the value of *s* with allele frequency in ancestral populations and in descendant populations
